# Regulatory Networks Controlling Neurotoxin Synthesis in *Clostridium botulinum* and *Clostridium tetani*

**DOI:** 10.3390/toxins14060364

**Published:** 2022-05-24

**Authors:** Michel R. Popoff, Holger Brüggemann

**Affiliations:** 1 Bacterial Toxins, Institut Pasteur, 75724 Paris, France; 2 Department of Biomedicine, Aarhus University, 8000 Aarhus, Denmark; brueggemann@biomed.au.dk

**Keywords:** *Clostridium tetani*, *Clostridium botulinum*, botulinum neurotoxin, tetanus neurotoxin, toxin gene regulation, two-component system, small RNA

## Abstract

*Clostridium botulinum* and *Clostridium tetani* are Gram-positive, spore-forming, and anaerobic bacteria that produce the most potent neurotoxins, botulinum toxin (BoNT) and tetanus toxin (TeNT), responsible for flaccid and spastic paralysis, respectively. The main habitat of these toxigenic bacteria is the environment (soil, sediments, cadavers, decayed plants, intestinal content of healthy carrier animals). *C. botulinum* can grow and produce BoNT in food, leading to food-borne botulism, and in some circumstances, *C. botulinum* can colonize the intestinal tract and induce infant botulism or adult intestinal toxemia botulism. More rarely, *C. botulinum* colonizes wounds, whereas tetanus is always a result of wound contamination by *C. tetani.* The synthesis of neurotoxins is strictly regulated by complex regulatory networks. The highest levels of neurotoxins are produced at the end of the exponential growth and in the early stationary growth phase. Both microorganisms, except *C. botulinum* E, share an alternative sigma factor, BotR and TetR, respectively, the genes of which are located upstream of the neurotoxin genes. These factors are essential for neurotoxin gene expression. *C. botulinum* and *C. tetani* share also a two-component system (TCS) that negatively regulates neurotoxin synthesis, but each microorganism uses additional distinct sets of TCSs. Neurotoxin synthesis is interlocked with the general metabolism, and CodY, a master regulator of metabolism in Gram-positive bacteria, is involved in both clostridial species. The environmental and nutritional factors controlling neurotoxin synthesis are still poorly understood. The transition from amino acid to peptide metabolism seems to be an important factor. Moreover, a small non-coding RNA in *C. tetani*, and quorum-sensing systems in *C. botulinum* and possibly in *C. tetani*, also control toxin synthesis. However, both species use also distinct regulatory pathways; this reflects the adaptation of *C. botulinum* and *C. tetani* to different ecological niches.

## 1. Introduction

*Clostridium botulinum* and *Clostridium tetani* are Gram-positive, spore-forming, and anaerobic rod-shaped bacteria that produce the most potent toxins among bacterial, animal, and plant toxins. *C. tetani* synthesizes the tetanus neurotoxin (TeNT), which is responsible for an often fatal spastic paralysis in humans and animals, whereas *C. botulinum* produces botulinum neurotoxins (BoNTs), which induce severe flaccid paralysis in vertebrates. Although TeNT and BoNTs lead to opposite clinical symptoms, they use a similar molecular mechanism of action. Indeed, TeNT and BoNTs deliver into target neurons their intracellularly active domain (light chain, Lc), which exerts a specific protease activity towards one of the three SNARE (soluble N-ethylmaleimide-sensitive factor attachment protein receptor) proteins: synaptobrevin or VAMP (vesicle-associated membrane protein), SNAP25 (synaptosomal-associated protein 25), and syntaxin. This leads to the inhibition of exocytosis of synaptic vesicles containing neurotransmitters. However, through their heavy chains (Hc), which recognize distinct cell surface receptors, TeNT and BoNTs undergo distinct trafficking in the host and target different neuronal cell types. Thereby, BoNTs target the motoneuron endings of the peripheral nervous system and block the release of acetylcholine, leading to a flaccid paralysis (botulism). In contrast, TeNT enters the central nervous system via retrograde transport through motoneurons and specifically inhibits the release of neurotransmitters (glycine, gamma aminobutyric acid (GABA)) in inhibitory interneurons, thus disrupting the negative control exerted by inhibitory interneurons over motoneurons, resulting in spastic paralysis (tetanus) [1,2].

## 2. Diversity of Clostridial Neurotoxins and Neurotoxigenic Bacterial Strains

BoNTs constitute a family of neurotoxins that share a similar structure and mode of action. They are classically divided into seven toxinotypes (A, B, C, D, E, F, and G) based on their neutralization by corresponding specific polyclonal antibodies. At the genetic level, two additional types, H or F/A, and X, have been described [3,4]. Each BoNT type is subdivided into subtypes based on amino acid sequence variations (0.9–36.2%, in most cases >2.6%). More than 40 subtypes have been identified [5]. Most BoNTs are produced by *C. botulinum* strains that are classified into physiologically and genetically distinct groups: group I including proteolytic strain types A, B, F; group II including non-proteolytic strain types B, E, F; group III corresponding to *C. botulinum* types C and D; and group IV, referred to as *Clostridium argentinense* type G. BoNTs are also synthesized by some other clostridial species, such as BoNT/E by atypical *Clostridium butyricum* strains and BoNT/F by atypical *Clostridium baratii* strains [5]. Moreover, a *Paraclostridium bifermentans* strain produces a BoNT-like neurotoxin (PMP1), which is insect-specific, whereas BoNTs are only active in vertebrates [6]. Sequences related to *bont* genes have been found in the genomes of a few other non-clostridial bacteria, such as *bont*/Wo or *bont*/I from *Weisenella oryzae*, a bacterium isolated from fermented rice, *bont*/J (e*bont*/F or *bont*/En) in an *Enterococcus faecalis* strain isolated from a cow, and Cp1 in *Chryseobacterium piperi* isolated from sediment [7,8,9,10,11,12]. However, the production of BoNT by non-clostridial strains has not been reported and their activity remains to be characterized.

In contrast to the heterogeneity of BoNTs- and BoNT-producing clostridia, TeNT is highly conserved [13,14]. Only a few amino acid variations were observed, notably a four-amino-acid insertion in four strains, in a study that investigated 37 *C. tetani* strains [13]. TeNT-producing *C. tetani* strains show a high level of genomic conservation. The population of *C. tetani* is divided into two major closely related clades and most strains belong to clade 1 [13].

## 3. Genetic Organization of Clostridial Neurotoxin Genes

**Figure 1:**
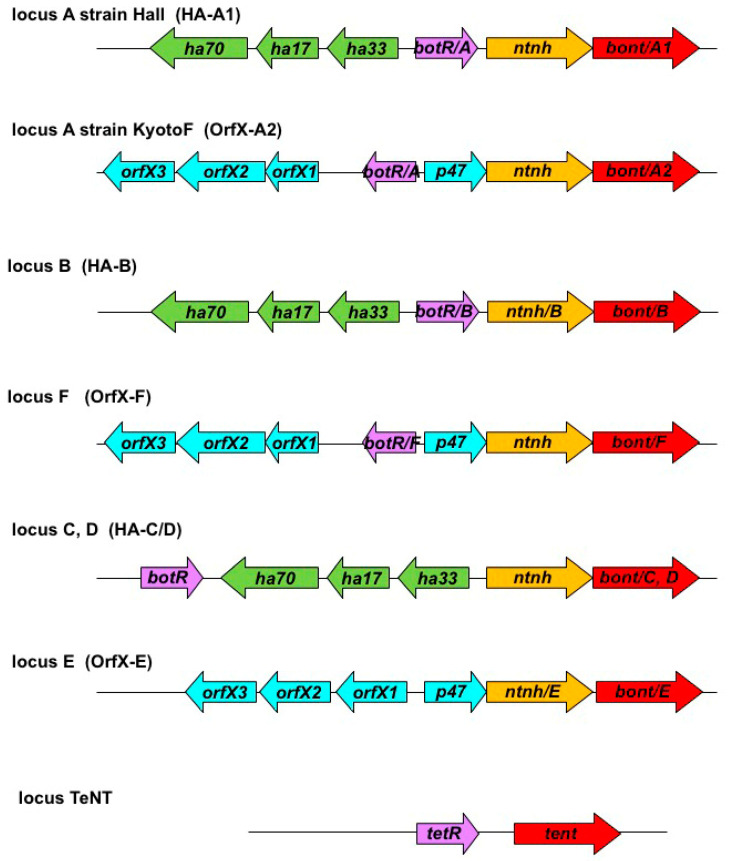
Genetic organization of neurotoxin genes in representative *Clostridium botulinum* and *Clostridium tetani* strains. red, neurotoxin genes; orange, *ntnh* genes; purple, regulatory genes; green, *ha* genes; cyan, *orfX* and *p47* genes.

BoNT-producing clostridia synthesize non-toxic proteins (associated-non-toxic proteins, ANTPs), which assemble with BoNTs through non-covalent bounds to form large-sized botulinum complexes (also referred to as progenitor toxins). A main characteristic of botulinum complexes is that they are stable at acidic pH and dissociate at alkaline pH (≥7) into free BoNT and ANTPs [15]. The genes encoding BoNTs and ANTPs are clustered in a DNA fragment called the botulinum locus. The genetic organization of the *bont* locus was initially determined in *C. botulinum* type C, where it is localized on a phage [16]. It consists of two operons transcribed in opposite directions. One operon contains *bont* and, located immediately upstream, *ntnh*, which encodes for the non-toxic non-hemagglutinin (NTNH) protein. NTNH shares a similar size and structure with BoNT, but NTNH lacks the catalytic site of BoNT. The interlocked association of NTNH with BoNT confers high resistance to acidic and protease degradation, while each protein separately is sensitive to proteolysis [17]. The *ntnh-bont* operon is highly conserved in all BoNT-producing clostridia, suggesting that *ntnh* and *bont* derive from a common ancestor by gene duplication [18,19,20]. The second operon is more divergent; it contains either hemagglutinin (HA) genes, including *ha70*, *ha17*, and *ha33*, or *orfX* genes (*orfX1, orfX2, orfX3*). The botulinum loci with *orfX-bont* genes encompass an additional gene in the first operon, *p47*, located upstream of *ntnh* [19,21]. A gene, *botR*, encoding an alternative sigma factor is localized in the botulinum loci upstream of the two operons in *C. botulinum* C and D and between the two operons in the other *C. botulinum* types, but it is lacking in *C. botulinum* E (Figure 1) [22,23]. HA complexes have been found to facilitate the passage of BoNT through the intestinal barrier by disrupting E-cadherin intercellular junctions between intestinal epithelial cells [24,25,26]. Up to now, no function has been attributed to OrfX and P47 proteins.

In *C. tetani*, the *tent* gene is localized on large plasmids. In comparison to the chromosome, these plasmids exhibit a higher degree of diversity. A homologous gene to *botR*, called *tetR*, lies immediately upstream of *tent.* No genes related to *C. botulinum* ANTP genes were identified in *C. tetani* genomes (Figure 1) [13,27,28].

## 4. Alternative Sigma Factors

BotR and TetR are the first factors that have been identified to be involved in the regulation of neurotoxin synthesis in *C. botulinum* and *C. tetani.* Overexpression of *bot/R* and *tetR* in *C. botulinum* and *C. tetani*, respectively, enhances neurotoxin synthesis (approximately 10-fold based on overexpression with a multicopy vector; toxin levels were quantified using toxin titration by mouse lethal activity), and inversely, their partial repression reduces toxin production (approximately 10-fold, based on experiments using an antisense mRNA strategy) [22,27]. Indeed, BotR and TetR are positive regulators of neurotoxin synthesis, and BotR also regulates the HA production in *C. botulinum* A [22]. Interestingly, overexpression of *botR* from *C. botulinum* type C in *C. tetani* stimulates TeNT synthesis, indicating a common mechanism of action of BotR and TetR [27]. BotR/A from *C. botulinum* A binds to conserved motifs in the two promoters of both operons, *ntnh-bont/A* and *ha*, in an RNA polymerase core enzyme-dependent manner. These motifs are also conserved in the promoter of *tent* and facilitate the binding of the TetR-RNA polymerase core enzyme complex. Thereby, BotR/A and TetR drive the transcription of the corresponding neurotoxin genes, and in addition, BotR/A controls that of the *ha* genes in *C. botulinum* [29]. Homologs of BotR and TetR have been characterized in *Clostridioides difficile* and *Clostridium perfringens*, where they positively control toxin synthesis. TcdR from *C. difficile* regulates the production of Toxin A (TcdA) and Toxin B (TcdB), and UviA controls the synthesis of a *C. perfringens* bacteriocin. BotR, TetR, TcdR, and UviA, which are interchangeable, as tested by in vitro transcription, are assigned to a distinct subgroup (group 5) of alternative sigma factors based on their targeted DNA motifs [30,31].

BotR and TetR are concomitantly expressed with their corresponding neurotoxin genes, showing a maximum level of expression at the end of the exponential growth phase and beginning of the stationary phase. The transcription levels of *botR* or *tetR* are approximately 100-fold less than those of *ntnh-bont*, *has*, or *tent*, respectively, as it is usually observed between regulatory and target genes [32,33]. Levels of neurotoxins secreted into the culture supernatant follow a similar pattern, including a progressive accumulation during the exponential growth, reaching a maximum level at the beginning of the stationary phase and maintaining a stable toxin level. Since *botR* and *tetR* are located in close proximity to neurotoxin and ANTP genes, they primarily have an impact on these target genes. However, *botR* and *tetR* might also regulate other distantly located genes, as is observed with most of the regulatory genes that have widely pleiotropic effects. Indeed, in *C. botulinum* A, *botR* seems to control the expression of at least 21 genes, 15 and 6 being up- and downregulated, respectively [34]. In contrast to primary sigma factors that are required for controlling house-keeping genes, alternative sigma factors are involved in controlling growth phase transitions, such as from the exponential growth to stationary phase, in response to environmental factors, including nutritional factors and stress conditions such as oxidative stress or heat shock. Their regulatory activity also comprises morphological differentiation, flagellar biosynthesis, and sporulation. In most cases, environmental bacteria that are exposed to a wide range of external factors employ more sigma factors than obligate pathogens or commensals, which are adapted to a specific host compartment and have a more restricted environment. Many obligate pathogens have lost sigma factors after host adaptation, such as *Mycobacterium leprae*, a strictly obligate pathogen that contains four sigma factors, compared to 13 sigma factors in *Mycobacterium tuberculosis*, which can live in the environment [35,36]. Indeed, *C. botulinum* A and *C. tetani* possess 18 and 25 sigma factors, respectively [37], compared to seven in *Escherichia* coli K12, six in *Shigella flexneri*, and three in *Helicobacter pylori*, the two latter species being strict human pathogens [38]. Group 4 alternative sigma factors, also called sigma factors of the extracytoplasmic function (ECF) family, are mainly involved in sensing and responding to extracellular signals and regulate cell envelope functions (transport, secretion, bacterial cell wall stress response) [31,36,39]. Group 5 alternative sigma factors, including BotR and TetR, are distantly related to the other sigma factors based on amino sequence similarity [30,31,36]. They control toxin synthesis and possibly other bacterial functions in response to yet unknown environmental stimuli or conditions.

In group III *C. botulinum* strains, the *bont* locus is located on a phage, and, in contrast to group I and II *C. botulinum* strains, where *botR* lies between the two *has/orfX* and *ntnh-bont* operons, *botR/C* or *D* is upstream of both *has* and *ntnh-bont* operons. The binding motifs recognized by BotR/A in the promoters of the *has* and *ntnh-bont* operons in *C. botulinum* A are conserved in the corresponding promoter regions of *C. botulinum* C and D [29]. BotR/C and D play likely a similar regulatory role of *has* and *ntnh-bont* operons as in *C. botulinum* A, but possibly do not respond to the same environmental stimuli, since group III *C. botulinum* strains have different physiological properties—for example, a higher optimal growth temperature; they are more often associated with animals than group I and II *C. botulinum* [40,41,42].

Despite the fact that *C. botulinum* E strains lack *botR* in close proximity to *bont/E*, the kinetics of growth, *bont/E* expression, and BoNT/E production are similar to those in *C. botulinum* A and proteolytic and non-proteolytic strains of *C. botulinum* B [33,43,44], supporting the notion that additional regulatory genes are involved in the control of neurotoxin genes. The non-proteolytic *C. botulinum* E, F6, non-proteolytic *C. baratii* F7, and non-proteolytic *C. butyricum* E contain an *orfX-p47-ntnh-bont* locus without *botR*. However, *botR* is present just upstream of the *ha* operon in non-proteolytic *C. botulinum* B4 and *C. argentinense*, which both have a *ha-ntnh-bont* botulinum locus, similar to *C. botulinum* C and D. In *C. baratii* F7, a gene encoding an UviA-like protein belonging to the same subgroup of sigma factors as BotR lies upstream of the *orfX* operon. This gene is also found in non-proteolytic *C. botulinum* E and F6, but distantly located of the botulinum locus [45]. The UviA-like protein in non-proteolytic strains might have a similar regulatory function of botulinum locus genes to BotR.

## 5. Two-Component Systems

Two-component systems (TCSs) consist of two proteins which coordinately control gene transcription in response to extracellular signals. TCSs regulate various bacterial physiological processes required for adaptation to environmental changes, such as development, cell division, metabolism, pathogenicity, and antibiotic/bacteriocin resistance. One component is a transmembrane protein with an extracellular sensor domain and, more rarely, a cytoplasmic domain, called sensor histidine kinase (SHK), which senses environmental stimuli including small molecules, ions, toxics, dissolved gases, pH, temperature, osmotic pressure, redox potential, or other yet unknown factors. SHK communicates with a corresponding response regulator (RR) by a phosphorelay. Most of the SHK and RR genes are closely located and organized in operons. Signal sensing by the N-terminal region of SHK induces the phosphorylation of a conserved histidine (His) in the C-terminal part. SHKs are in dimeric form and retain the phosphorylated His in a dimeric helical domain. The His phosphoryl group is then transferred to a conserved aspartate residue in the receiver domain of the corresponding RR. This results in a conformational change of the RR and enhanced affinity for specific promoter(s) [34,46,47,48].

**Figure 2:**
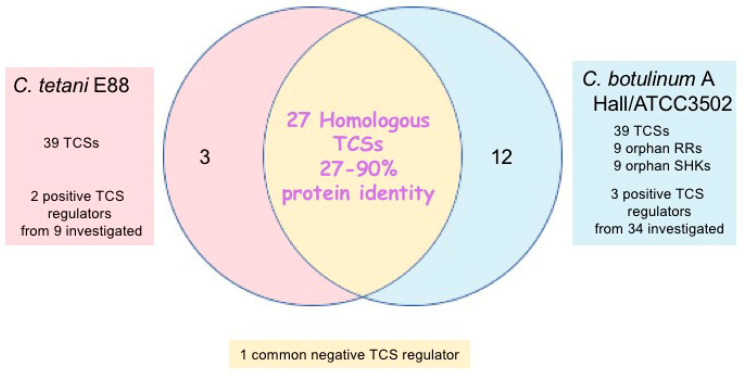
Two-component systems (TCSs) involved in neurotoxin gene regulation in *Clostridium botulinum* strain Hall and ATCC3502 and in *Clostridium tetani* strain E88.

*C. botulinum* and *C. tetani* contain numerous TCS genes in their genomes. In *C. botulinum* strain Hall, 39 genes have been found to encode RR proteins based on the presence of conserved motifs such as the signal receiver domain and DNA binding domain. Thirty RR genes are located in close proximity to a SHK gene, thus corresponding to 30 TCS operons. An additional nine RR genes and nine SHK genes are considered as orphan regulators [34,37]. *C. tetani* strain E88 possesses 30 TCS genes, 19 of which are homologous to related genes of *C. botulinum* A strain Hall, with ≥45% identity at the amino acid level (Figure 2) [32]. Most of the *C. botulinum* and *C. tetani* TCS genes have homologs in other clostridia, indicating that these clostridia might share similar regulatory pathways [32,34].

In *C. botulinum* A strain Hall, 34 regulatory genes (29 TCSs and five RR orphan regulatory genes) and an additional TCS gene in strain ATCC3502 (a derivative of strain Hall), as well as nine TCS genes in *C. tetani* E88, have been investigated for their possible regulatory contribution in toxinogenesis [32,34,49]. In *C. botulinum* A strain Hall, three TCSs have been reported as positive regulators of BoNT synthesis in a *bontR*-independent manner (as tested by an antisense mRNA strategy, yielding recombinant strains with 10- to 100-fold decreased BoNT/A production, which was determined by ELISA) [50]. Among them, one TCS belonging to the OmpR family shows 65% protein identity with a *C. tetani* TCS and shares similarity with VirI/VirJ of *Clostridium perfringens*, which has been reported as a regulator of toxin synthesis (GenBank BAA78773, BAA78774). Although this TCS is a positive regulator of BoNT synthesis, the *C. tetani* homolog is not involved in TeNT synthesis. The two other TCSs that are positive regulators of BoNT synthesis have no homolog in *C. tetani.* Two additional TCSs in *C. botulinum* show an indirect effect on BoNT production as they have pleiotropic effects, notably by impairing cell wall synthesis or assembly. The two *C. tetani* TCSs that positively regulate the TeNT synthesis (as tested by an antisense mRNA strategy, yielding recombinant strains with two- to five-fold decreased TeNT production, which was determined by ELISA) have ineffective homologs in BoNT synthesis in *C. botulinum* A [32] (Table 1). Only one TCS that is conserved (100% protein identity) in *C. botulinum* A strain ATCC3502 and in *C. tetani* E88 is a negative regulator of neurotoxin synthesis in both microorganisms (as evaluated by the ClosTron (http://www.clostron.com (accessed on 13 April 2022), Nottingham, UK) strategy in strain ATCC3502, where BoNT/A levels were monitored by ELISA; in strain E88, the antisense mRNA strategy was applied and TeNT levels were determined by ELISA) [32,49]. In *C. botulinum*, this TCS binds to both promoters of the *ntnh-bont* and *ha* operons and prevents their transcription by impairing the binding of BotR [49]. In *C. tetani*, the corresponding TCS binds also to the *tent* promoter and likely retains the same mechanism of action as in *C. botulinum* ATCC3502. However, the apparent TCS counterpart in *C. botulinum* strain Hall, which shows only 58% protein identity, is apparently not involved in the regulation of BoNT synthesis, as judged from the lack of BoNT level alterations assayed by ELISA in the recombinant Hall strain using the anti-sense RNA strategy targeting this TCS (Table 1) [32,50]. Thereby, various TCSs control the neurotoxin synthesis in *C. botulinum* and *C. tetani.* Although *C. botulinum* and *C. tetani* share homologous TCSs, most of them have distinct functional roles in the control of neurotoxin synthesis in the two microorganisms.

## 6. Metabolism and Toxin Gene Regulation

Toxin synthesis, as with protein synthesis in general, is dependent on the metabolic activity of the bacteria. How is neurotoxin synthesis linked to the general metabolism?

### 6.1. CodY

In Gram-positive bacteria, CodY (control of dciA (decoyinine induced operon) Y) is a master regulator of metabolism, sporulation, and virulence. In *Bacillus subtilis*, CodY controls more than 100 genes involved in the adaptation to nutrient restriction and transition from the exponential growth phase to the stationary growth phase. Typically, CodY acts by binding to the promoter of target genes in a GTP(guanosine triphosphate)- and branched-chain amino acid-dependent manner; these are indicators of the general metabolism status of the bacterium [51]. Thereby, in *B. subtilis*, CodY senses intracellular levels of GTP and branched amino acids such as isoleucine, whose levels are high during the exponential growth and decrease in mostly repressed gene transcription. In contrast, at low GTP or isoleucine levels, CodY induces the de-repression of genes which are involved in adaptive responses to nutrient limitation, such as those coding for extracellular degradative enzymes, transport systems, and catabolic pathways [51,52]. CodY is conserved in clostridia, including the toxigenic species *C. botulinum*, *C. tetani*, *C. perfringens*, and *C. difficile*. In *C. botulinum* A strain ATCC3502, CodY binds to the promoter of the *ntnh-bont* operon at high GTP levels, whereas isoleucine is ineffective, and stimulates toxin gene transcription and BoNT/A synthesis (as tested with the ClosTron system, and determining BoNT/A levels by ELISA and *bont/A* transcription by qPCR) [53]. The precise role of CodY in *C. botulinum* is still elusive: does CodY directly regulate *ntnh-bont* transcription or interfere with *botR* or a TCS gene such as by repressing the negative TCS regulator? CodY is also a positive regulator of TeNT synthesis in *C. tetani* (as tested by the antisense mRNA strategy, and determining TeNT levelsby ELISA, and *tent* transcription by qPCR) [32]. CodY binds to the *tent* promoter but not to that of *tetR* [32]. BoNT and TeNT synthesis is dependent on the availability of a carbon source such as glucose [54,55,56]. CodY controls carbon metabolism in *B. subtilis.* Notably, under glucose-rich conditions in culture medium, CodY and CcpA (catabolite control protein A), a regulator of carbon catabolism, facilitate the conversion of excess pyruvate resulting from glycolysis into excretable overflow compounds such as acetate, lactate, and acetoin [57]. A similar mechanism of CodY in glucose/pyruvate metabolism has been suggested in *C. botulinum* A [53]. CcpA is conserved in *C. botulinum* and *C. tetani.* However, the role of CcpA in these microorganisms remains to be elucidated. In contrast, in *C. difficile*, CodY and CcpA are negative regulators of toxin A (TcdA) and toxin B (TcdB). Glucose and rapidly metabolizable carbohydrates inhibit toxin synthesis in *C. difficile.* CodY and CcpA, which are activated by glucose and rapidly metabolizable carbohydrates, bind to the promoter of TcdR and repress its transcription and subsequently that of *tcdA* and *tcdB* [58,59,60]. The opposite regulatory pathways of toxin synthesis linked to carbohydrate metabolism controlled by CodY and CcpA between *C. botulinum/C. tetani* and *C. difficile* are intriguing. This indicates that toxin synthesis in *C. botulinum* and *C. tertani* requires energy from carbohydrate metabolism, mainly glucose, the main carbohydrate fermented by these bacteria, while *C. difficile* mainly uses amino acid metabolism as an energy source, notably through the Stickland reaction, for toxin production [60,61,62,63]. These divergent regulatory pathways might have evolved during bacterial adaptation to different environments: soil for *C. botulinum/C. tetani* and the intestine for *C. difficile.*

### 6.2. Spo0A

Spo0A is a master regulator of the initial steps of sporulation in *Bacillus* and clostridia. However, the mode of activation of Spo0A differs in the two classes of bacteria. Nutrient limitation, notably carbohydrate, nitrogen, and phosphorus limitation, is the major signal leading to Spo0A activation through a phosphorelay including five sensor kinases and subsequent positive transcriptional regulation of critical sporulation-essential genes [64]. The kinases that activate Spo0A in *Bacillus* are not conserved in clostridia. Orphan Spo0A-activating histidine kinases have been identified in clostridia, such as *Clostridium acetobutylicum*, *C. perfringens*, *C. difficile*, *C. botulinum*, and *C. tetani*. Clostridia sense different environmental stimuli to initiate sporulation, including external pH resulting from fermentation, with the subsequent release of acidic end-products (acetate, butyrate) and unknown factors [65,66,67,68,69]. In clostridia, Spo0A displays additional functions apart from sporulation initiation. In *C. acetobutylicum*, Spo0A is activated at the end of the exponential growth phase and controls the shift between acidogenesis that occurs during the exponential growth, and solventogenesis that is coupled to the onset of sporulation [70,71]. In *C. perfringens*, Spo0A controls the production of toxins (*C. perfringens* enterotoxin and TpeL), which are synthesized during the sporulation process [72,73]. The role of Spo0A in the regulation of TcdA and TcdB synthesis is variable according to the genetic background of *C. difficile* strains [62,74]. Spo0A coordinates the expression of a large number of *C. difficile* genes involved in multiple additional functions, such as nutrient transport, metabolic pathways including the production of butyrate, surface protein assembly, and flagellar biosynthesis [75].

Spo0A is highly conserved in all *C. botulinum* genomes: an orphan sensor histidine kinase that is able to phosphorylate Spo0A has been identified [69]. In *C. botulinum* ATCC3502, Spo0A is expressed during the exponential growth and its expression decreases during the entry into the stationary phase, while the subsequent transcription of sigma factors essential for sporulation increases [76,77]. It is not known whether Spo0A affects the expression of *bont* in group I *C. botulinum*. Adaptation to cultivation at high temperatures (45 °C) represses both *bont*/A and sporulation genes in strain ATCC3502, but no co-regulation of these genes has been evidenced [78]. No correlation between sporulation and the production of BoNT/A has been observed in two other *C. botulinum* A strains [33]. Moreover, the strain Hall A-*hyper* produces high levels of BoNT/A and is unable to sporulate [79]. Similarly, the highly TeNT-producing *C. tetani* strain used for vaccine production is a non-sporulating strain [80], and Spo0A has not been found to control TeNT synthesis (as tested by the antisense mRNA strategy, TeNT monitoring by ELISA, and *tent* transcription by qPCR) [32]. In contrast, in group II *C. botulinum* E, Spo0A is a positive regulator of BoNT/E synthesis and sporulation (as tested with the ClosTron system, toxin monitoring by ELISA, and gene transcription by qPCR). Spo0A binds to a conserved motif in the promoters of the *ntnh-bont/A* operon together with CodY, AbrB (putative repressor of *bont/E*), sigma K (belonging to the sigma factor cascade of sporulation), and an UviA-like regulator [43]. Thus, Spo0A might directly and indirectly regulate the transcription of *bont/E.* Thereby, group II *C. botulinum* E strains that have an UviA-like regulator instead of BotR likely use specific and common regulatory pathways of *bont* expression compared to *C. botulinum* A strains which belong to the distinct physiological and genetic group 1.

In group III *C. botulinum* C and D, the production of the C2 toxin, which is an ADP-ribosyltransferase targeting monomeric actin, is linked to sporulation [81]. However, the regulatory pathway of C2 toxin genes, and the possible involvement of *spo0A* and/or other sporulation genes, has not yet been elucidated.

### 6.3. Amino Acid/Peptide Metabolism

*C. botulinum* and *C. tetani* produce high levels of toxins in complex media rich in peptones and other nutrients, whereas chemically defined media even containing almost all amino acids and vitamins as well as a carbon source usually yield 10- to 100-fold lower toxin titers [55,82,83,84]. Licona-Cassani et al. showed that, although *C. tetani* grew in a chemically defined medium, toxin production was obtained only when casein-derived peptides were added to the medium [82]. In addition to variations in toxin production according to different media, variations in BoNT or TeNT yields are often observed from batch to batch of the same culture medium, even using the same bacterial strain. The transcription of neurotoxin genes and toxin synthesis occur mainly within a short time interval between the late exponential growth and early stationary growth phase [33,85,86]. Thus, nutritional and environmental factors influence the regulation of toxin synthesis in *C. botulinum* and *C. tetani*, which takes place in a restricted phase of bacterial growth. Peptides and amino acids appear to be important regulatory factors at the transcriptional and posttranscriptional levels. Indeed, large amounts (0.8–0.9 g/L) of amino acids (aspartate, glutamate, serine, histidine, threonine) downregulate *tetR* and *tent* by a yet non-identified regulatory pathway [86]. Arginine is an essential amino acid for *C. botulinum* growth, but an excess of arginine represses BoNT production in proteolytic group I *C. botulinum* [87]. Arginine deiminase leads to arginine catabolites that increase the pH and induce subsequent BoNT degradation by not yet characterized proteases. BoNT and botulinum complexes are stable at acidic pH in media without excess of arginine [88]. Supplementation with a high amount of glucose (50 g/L) that induces acidification counteracts the effect of arginine. Interestingly, BoNT synthesis is coupled to protease production [89]. Likely, proteases that are active at alkaline pH induce BoNT degradation. Secreted proteases are required for protein substrate degradation, resulting in peptides and amino acids that are taken up into the bacteria through transport systems and used for protein synthesis, including neurotoxin synthesis. Indeed, the *C. botulinum* A and *C. tetani* genomes contain numerous protease/peptidase and transport system genes [37,80].

Peptides in culture media were found to be critical for TeNT synthesis by *C. tetani.* Since culture media containing casein pancreatic digests support high levels of TeNT production, peptides derived from casein tryptic digestion were investigated. Histidine-containing peptides as well as hydrophobic peptides containing the motif proline–aromatic acid–proline were the most effective in promoting TeNT production [84,90,91]. It is noteworthy that genome analysis of *C. tetani* shows the presence of numerous peptidases and amino acid degradation pathways [92]. The kinetics of *C. tetani* growth in a complex medium show rapid exponential growth (stage I, around 10–12 h), then a slower linear growth (stage II, around 30 h), followed by a stationary phase and subsequent autolysis. During stage I, the amino acids are consumed and the genes involved in amino acid degradation pathways are overexpressed, corroborating amino acid catabolism that provides energy used for the rapid biomass formation during this growth phase. The pH decreases due to organic acid production, *tetR* and *tent* are not expressed, and TeNT is not synthesized. Once free amino acids are depleted in the culture medium, *C. tetani* uses peptides, whose metabolism requires transporters that are more energy-costly, and enters the linear growth phase II. The transition from free amino acid to peptide consumption is associated with increased pH due to the reduction of organic acids to alcohols and solvents, and the production of ammonia from peptide metabolism. During phase II, *tetR* and *tent* are highly expressed, as well as *codY*, the TCS that positively regulates *tent*, and two additional sigma factors located on the large plasmid containing *tent*, resulting in TeNT synthesis [54,82,86,93]. In complex media, glucose is consumed during the first phase of growth, leading to rapid bacterial multiplication and pH decrease. Then, the nitrogen source is used and the pH increases. TeNT is synthesized only during this second phase, when glucose is no longer or weakly available and when peptides are used for energy production. Thus, as shown by Fratelli et al., the balance between the nitrogen and carbon sources, as well as the subsequent pH of culture media, are critical factors [54,93]. *C. botulinum* and *C. tetani* likely share common metabolic pathways and subsequent toxin gene regulatory networks. In both microorganisms, the transition from amino acid utilization to peptides that are more common substrates in the environment seems to elicit the production of proteases. BoNT and TeNT are metalloproteases, but which recognize specific substrates in host neuronal cells. BoNT and TeNT possibly evolve from ancestor metalloproteases with a broader substrate range that were used by the bacteria for nutrient acquisition and that were regulated as the other proteases. Thus, the regulation of toxin synthesis in *C. botulinum* and *C. tetani* might represent a reminiscent common regulatory circuit controlling protease synthesis.

### 6.4. Other Nutritional and Environmental Factors

In addition to nutrients required for growth and protein synthesis, some nutritional and environmental factors might influence, directly or indirectly, toxin synthesis.

CO_2_—A high concentration of CO_2_ in the gas phase increases *bont* expression and BoNT synthesis in non-proteolytic group II *C. botulinum* B and E, although the growth rate is decreased. Indeed, a 70% CO_2_ atmosphere versus 10% stimulates 2- to 5-fold greater toxin gene expression and BoNT formation. In high and low CO_2_ concentrations, toxin gene expression occurs in the same growth phase, mainly in the late exponential growth and early stationary phase [94,95]. The signaling pathways in the regulation by CO_2_ are not known. CO_2_ can dissolve in the liquid medium and generate bicarbonate, which influences protein synthesis through carboxylation reactions. CO_2_ (35%) in the gas phase of *C. tetani* culture versus nitrogen atmosphere (unpublished) or the addition of sodium carbonate (100 mM) in the culture medium increases the production of TeNT approximately two-fold [32], despite reduced growth in the CO_2_-rich atmosphere (approximately three-fold). In contrast, elevated CO_2_ in the gas phase of proteolytic group I *C. botulinum* B and E has no effect on toxin gene expression [96], suggesting that CO_2_ triggers a signaling pathway controlling toxin synthesis in non-proteolytic strains.

Inorganic phosphate—Inorganic phosphate has been found to control TeNT synthesis in *C. tetani.* Supplementation of culture medium with inorganic phosphate (optimum concentration 40 mM) stimulates *tent* expression and TeNT production approximately three-fold without impairing the growth rate [32]. Inorganic phosphate is involved in multiple biochemical reactions; its effect on toxin gene transcription might be mediated by TCSs. *C. tetani* genome contains two TCSs putatively involved in phosphate uptake, one of which has been found to negatively regulate TeNT synthesis [32]. Inorganic phosphate is apparently not involved in BoNT production in *C. botulinum* A, as tested by supplementation of the TGY (trypticase-glucose-yeast extract) culture medium with 20 to 150 mM Na_2_HPO_4_ and monitoring BoNT/A production (strain Hall) in the culture supernatant by titration of the mouse lethal activity (unpublished). Control of the virulence mechanism by inorganic phosphate and TCS from the PhoP/PhoR family has been found in several pathogens [97,98]. TCSs control the homeostasis of phosphate according to the availability in the environment. However, the precise subsequent phosphate-dependent signaling pathways controlling virulence remain largely unknown.

pH—*C. botulinum* and *C. tetani* grow and produce the neurotoxins in a wide range of pH (pH 4.5–5 to 9). The initial pH of the growth medium was found to influence the autolysis of *C. tetani*. An initial pH of 6.1 seems optimal for TeNT production [84]. In a complex medium, high pH (7.8) downregulates *tent* [99]. The mechanism of toxin gene regulation by pH is not yet identified.

The culture pH of proteolytic *C. botulinum* A, B grown in complex media typically drops (pH 6–6.3 with initial glucose concentration up to 1%, and until pH 5.5 with glucose 1.5%) during the exponential growth phase, and then stabilizes and slightly increases during the stationary phase [56,85,88,100]. Maintaining an acidic pH (pH 5.7–6) during the culture does not modify the BoNT yield in the culture supernatant, whereas an alkaline pH (pH 7.2 and above, manually adjusted or by supplementation of the culture medium with 2% arginine) decreases the BoNT level [56,88]. The pH does not influence BoNT synthesis at the transcriptional level, but affects BoNT stability by activating a BoNT degrading metalloprotease in alkaline conditions [88].

Temperature—In contrast to *C. difficile*, in which a high temperature (42 °C) prevents *tcdR* and toxin gene expression, temperatures of 37–44 °C have no influence on *botR* and *bont* transcription in group I *C. botulinum* A. However, a high temperature induces the production of protease(s), which inactivate BoNT/A [33]. TeNT production is usually obtained by *C. tetani* culture at 33–35 °C [82,84,99].

Group II *C. botulinum* has an optimum temperature of 25 °C for growth and toxin production, but the strains of this group can grow and form toxins at temperatures as low as 3.0–3.3 °C in 5 to 7 weeks [101]. Investigation with *C. botulinum* E showed that growth and toxin production are lower at 10 °C than at 30 °C. However, *bontE* transcription relative to growth was similar at 10 °C and 30 °C [102]. A TCS is involved in the cold adaptation of *C. botulinum* E [103,104]. Similarly, cold tolerance of growth at 15 °C in *C. botulinum* A strain ATCC3502 requires the contribution of a TCS [105]. This TCS is conserved in *C. botulinum* A strain Hall, but it has not been identified as a regulator of BoNT/A synthesis [50]. Thus, temperature is important for growth and toxin production, but temperature seems to have no direct role in the regulation of toxin synthesis in *C. botulinum* and *C. tetani.*

## 7. Small RNA

In addition to regulatory proteins, bacteria use regulatory RNAs to modulate gene transcription or translation initiation for adaptive responses to environmental changes. Environmental bacteria have to adapt their physiology and metabolism to various hostile conditions and pathogenic bacteria have to cope with adverse host interactions; thus, they have to adapt rapidly their gene expression, notably that of virulence genes [106,107]. Regulatory RNAs are small molecules, typically between 50 and 500 nucleotides (small RNAs or sRNAs), and are non-protein coding. The main advantage of sRNAs versus regulatory proteins is their speed in controlling gene expression based on the faster availability of sRNAs due to the lower energy needed for their production by transcription and not by translation, as in the case of regulatory proteins, the faster turnover of sRNAs since RNAs are less stable than proteins, the rapid control of mRNA function by pairing with specific motifs in the untranslated region (UTR), or, in some cases, by acting at a posttranscriptional level [108,109]. Although most sRNAs are inhibitors of gene expression, some are activators. sRNAs lie in or overlap with the 5′ or 3′ UTRs of target genes, in intergenic regions, or in the opposite DNA strand and are transcribed as antisense sRNAs. Based on their genomic localization and mechanism of action, sRNAs are divided into several classes: *cis*-encoded sRNAs are transcribed from the DNA strand opposite to the target sequence and interact by perfect base pairing with mRNAs; *trans*-encoded sRNAs are distantly transcribed from target mRNA genes and recognize their target mRNAs by multiple and discontinuous short contacts. Clustered, regularly interspaced short palindromic repeat (CRISPR) RNAs interact with foreign DNA or RNA. Another class of sRNAs bind to regulatory proteins and antagonize their function. RNA riboswitches sense metabolites or environmental cues; they are usually located in the 5′ UTR of their target genes. Certain sRNAs interact with regulatory proteins, notably by promoting protein sequestration, but most sRNAs interfere with mRNA by inhibition of their translation, such as by blocking the ribosome binding site and/or by impairment of mRNA stability, resulting in subsequent degradation [108,109,110,111]. Numerous sRNAs act at a posttranscriptional level, whereas regulatory proteins preferentially act at transcriptional steps. In fact, numerous bacterial gene regulations involve mixed regulatory networks, including both transcriptionally acting regulatory proteins and posttranscriptionally acting sRNAs [112].

sRNAs are widespread in Gram-positive bacteria and notably in clostridia. In environmental clostridia such as *C. acetobutylicum*, sRNAs (159 predicted) have a crucial role in solventogenesis, growth, and the response to toxic metabolites [113,114,115]. In pathogenic clostridia, sRNAs have been described in *C. perfringens* to be involved in toxin production, as well as in *Clostridioides difficile* in adaptation to host and anti-phage defenses [116,117,118]. More than 200 sRNAs are predicted in the genomes of groups I and II *C. botulinum*, and 137 in the genome of *C. tetani* E88 [119]. A sRNA has been identified in the 3′ UTR of *tent* in the *C. tetani* E88 strain that is conserved in all toxigenic *C. tetani* strains [120]. However, no such sRNA has been reported in *C. botulinum*. This sRNA is expressed concomitantly with *tent* and negatively regulates *tent* expression and TeNT synthesis. The sRNA (approximately 140 nucleotides) contains a predicted junction-loop-exposed 14-nucleotide-long sequence that perfectly matches to a complementary sequence in the 5′ region of *tent* mRNA. Thus, the sRNA-mediated inhibitory regulatory activity is likely based on the sequestration of *tent* mRNA. In addition, this sRNA impairs *C. tetani* growth, notably by reducing the exponential growth phase [120]. Pleiotropic effects of sRNAs have also been found for the regulatory RNA VR-RNA of *C. perfringens* that controls 147 genes, including genes of toxins and virulence factors (alpha-toxin, kappa-toxin, hyaluronidase, sialidases) as well as genes involved in capsule synthesis [116,121].

## 8. Quorum Sensing

Quorum sensing is a cell-to-cell communication that bacteria use to adapt their physiology and behavior in response to cell densities. Bacteria produce and secrete extracellular signaling molecules called autoinducers. When a certain threshold of bacterial density is reached, accumulated autoinducers are detected by the bacteria, leading to coordinated changes in gene expression and behavior. Thus, quorum sensing allows a synchronized adaptation to environmental conditions in nonclonal bacterial populations [122,123]. A well-characterized quorum-sensing system is the Agr system of *Staphylococcus aureus* [124]. The autoinducer is a small peptide (autoinducing peptide, AIP) produced by *agrD* as a precursor, which is processed by the membrane endoprotease AgrB. When AIP reaches a threshold concentration in the environment, it is sensed by the TCS AgrA/AgrC, which in turn upregulates the expression of a small RNA and subsequently stimulates toxin synthesis. AgrC is the histidine kinase cell surface receptor that recognizes AIP and activates the response regulator AgrA [122]. Quorum sensing has been evidenced in *C. botulinum* [125], and an Agr-like system has been identified in group I *C. botulinum* strains called *agr-1/agr-2*; *agr-1* and *agr-2* are homologs of *agrB*, and *agrD*, respectively [126]. However, the Agr system in *C. botulinum* plays a different role than in *S. aureus*. Agr-1 seems to be involved in sporulation, while Agr-2 possibly controls toxin production [126]. Homologous genes of *agrA* and *agrC* have also been identified in *C. botulinum* A Hall strain, but silencing *agrA* did not impact BoNT/A production [34]. Thus, the regulatory quorum-sensing pathways in *C. botulinum* remain to be defined. A computational model of group I *C. botulinum* A growth and toxin production based on nutrient availability, cell density, and quorum-sensing signaling has been proposed in agreement with experimental data [127].

It is noteworthy that a cultivation technique was developed in the 1950s and 1960s to obtain high levels of toxin [128]. This method consists of dialyzed cultures. The bacteria are inoculated inside a dialysis bag (usually a cellophane bag) containing saline, which is immersed in a culture medium. This technique was used for the production of BoNT and TeNT, with a 5–50-fold increase in toxin yields compared to cultures in a flask with the same culture medium [128,129,130,131]. A very high bacterial density is obtained by culturing clostridia in dialysis bags and likely a quorum-sensing-mediated regulation is involved in the production of high toxin levels. Moreover, the high bacterial density leads to increased autolysis, which contributes to toxin release into the extracellular medium.

## 9. Concluding Remarks

**Figure 3:**
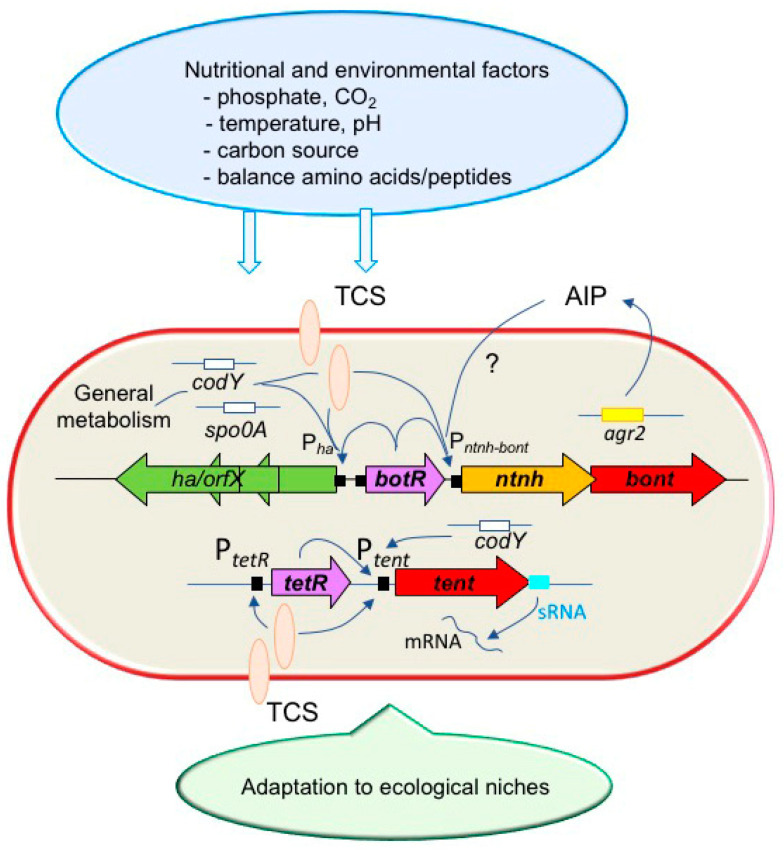
Schematic representation of the regulatory pathways in *Clostridium botulinum* and *Clostridium tetani.* TCS, two-component system; AIP, autoinducing peptide. External factors act through TCSs and/or other unknown receptors or transporters (blue arrows).

The toxigenic environmental bacteria *C. botulinum* and *C. tetani* contain multiple and complex regulatory networks to control neurotoxin production. These only partially deciphered networks include alternative sigma factors, TCSs, sRNAs, quorum-sensing systems, and regulators of bacterial metabolism that interlock the bacterial growth with toxin production (Figure 3). Both microbial species share some common regulatory mechanisms, notably an alternative sigma factor, the gene of which is located upstream of the neurotoxin gene, and an inhibitory TCS. They retain a similar kinetic pattern of toxin production, mainly occurring at the transition between the exponential growth and early stationary growth phases. This is possibly linked to the alternative sigma factors BotR and TetR, which are expressed concomitantly with the neurotoxin genes. However, *C. botulinum* and *C. tetani* use distinct signaling pathways, notably distinct TCSs, probably reflecting the recognition of different nutritional and/or environmental signals. Although toxinogenesis is dependent on the general metabolism in *C. botulinum* and *C. tetani*, the nutritional requirements for toxinogenesis seem different between these microorganisms. However, only a few nutritional and environmental factors controlling the toxinogenesis have so far been identified, such as CO_2_ in group II *C. botulinum* and inorganic phosphate in *C. tetani*. Differences in nutritional factors, particularly in the nature and composition of peptides and amino acids required for toxinogenesis, seem a major hallmark between *C. botulinum* and *C. tetani.* This likely reflects the different ecological niches used by these bacteria. Group I *C. botulinum* prefers neutral to slightly alkaline soils with low organic content, while group II *C. botulinum* are mostly found in more acidic soils with high levels of organic matter or as commensals in the intestines of certain animals. *C. tetani* is found primarily in neutral or alkaline soils at sufficient temperatures (>20 °C) and levels of moisture (15%) [41,132]. Moreover, group I but not group II *C. botulinum* strains can colonize the intestines of humans and produce BoNT in situ, leading to infant botulism or adult intestinal toxemia botulism [133]. *C. tetani* has not been reported to colonize the digestive tract and to induce intestinal tetanus [134]. Thus, *C. botulinum* groups and *C. tetani* have adapted to particular environments, notably through complex and specific regulatory systems that sense extracellular signals, leading to adapted gene expression. In addition to regulatory proteins and sRNAs, clostridia sense environmental factors by specific arrays of surface-associated proteins [135]. Do BoNTs and TeNT represent adaptive factors? These neurotoxins that attack specifically the nervous systems of vertebrates seem not to be involved in environmental adaptation. Indeed, non-toxigenic strains of *C. botulinum* and *C. tetani* can multiply, sporulate, and survive in the environment in the same manner as their toxigenic counterparts. BoNTs and TeNT, which likely evolved from a common protease ancestor, possibly retain common regulatory mechanisms with other proteases/peptidases required for the utilization of specific nutrient sources [20,136]. This is further supported by the observation that toxin synthesis is initiated at the transition from amino acid/carbohydrate to peptide metabolism in *C. Tetani* [54,93], and possibly in *C. botulinum.*

## Figures and Tables

**Table 1:** Two-component systems involved **in toxin** gene regulation in *C. botulinum* Hall and *C. tetani* E88.

*C. tetani* E88						*C. botulinum* Hall			
Genetic Localization	Locus Tag	Role	Family RR	Regulation of TeNT Synthesis	Ref.	Homolog	Protein Identity (RR)	Regulation of BoNT Synthesis	Refs.
chr	CTC_RS10150 CTC_RS10155	SHK RR	LytR/AlgR	Positive	[32]	CLC_3250 CLC_3251	55%	None	[50]
plasmid	CTC_RS13810 CTC_RS13805	SHK RR	OmpR	Positive	[32]	CLC_1431 CLC_1432	56%	None	[50]
	No homolog		OmpR			CLC_1093 CLC_1094		Positive	[50]
	No homolog		OmpR			CLC_1913 CLC_1914		Positive	[50]
chr	CTC_RS02080 CTC_RS02085	RR SHK	OmpR	None	[32]	CLC_0661 CLC_0663	65%	Positive	[50]
chr	CTC_RS10030 CTC_RS10035	SHK RR	OmpR	None	[32]	CLC_0410 CLC_0411	68%	Cell wall alteration	[50]
	No homolog		OmpR			CLC_3293 CLC_3294		Cell wall alteration	[50]
chr	CTC_RS07310 CTC_RS07315	SHK RR	OmpR	Negative	[32]	strain Hall CLC_0842 CLC_0843	58%	None	[50]
						strain ATCC3502 CBO_0786 CBO_0787	100%	Negative	[49]

chr, chromosome; RR, response regulator; SHK, sensor histidine kinase. positive effects are in green, and negative effects are in pink.

## Data Availability

Not applicable.
